# Comparative analyses on nitrogen removal microbes and functional genes within anaerobic–anoxic–oxic and deoxidation ditch sewage-treating processes in Wuhan and Xi’an cities, China

**DOI:** 10.3389/fmicb.2024.1498681

**Published:** 2024-10-30

**Authors:** Shuangyuan Liu, Yaqi Liu, Linyan Ye, Enrong Xiao, Dong Xu, Hongjun Chao, Jingcheng Dai, Dongru Qiu

**Affiliations:** ^1^School of Life Science and Technology, Wuhan Polytechnic University, Wuhan, China; ^2^Institute of Hydrobiology, Chinese Academy of Sciences, Wuhan, China; ^3^Eco-Environmental Monitoring and Research Center, Pearl River Valley and South China Sea Ecology and Environment Administration, Ministry of Ecology and Environment, Guangzhou, China; ^4^Ecological Environment Science and Technology Center, Wuhan, China

**Keywords:** anaerobic–anoxic–oxic system, deoxidation ditch process, nitrogen metabolism genes, seasonal variation, wastewater treatment

## Abstract

**Introduction:**

Anaerobic–anoxic–oxic (A^2^/O) and deoxidation ditch (DOD) processes are being increasingly preferred owing to their effectiveness in treating various wastes in wastewater treatment plants (WWTPs). Considering seasonal variations is crucial in optimizing treatment processes, ensuring compliance with regulations, and maintaining the overall efficiency and effectiveness of WWTPs. This study aimed to determine the influence of seasonality on nitrogen removing microbes and functional genes within A^2^/O and DOD processes in the humid Wuhan and semi-arid Xi’an cities, China.

**Methods:**

The physicochemical parameters of water quality were determined, and molecular and bioinformatic analyses of the bacterial community and nitrogen metabolism functional genes in the two different treatment processes of two WWTPs were performed over four seasons.

**Results and discussion:**

Our analyses revealed a significant difference in all physicochemical parameters across all experimental groups (*p* < 0.05). At the genus level, the abundance of *Dokdonella*, one unidentified genus of *Nitrospiraceae*, *Terrimonas*, and one unidentified genus of *Chloroflexi* was the highest in all groups. Generally, warmer seasons exhibited higher biodiversity indices. The A^2^/O system exhibited higher values in terms of most nitrogen metabolism functional genes than those of the DOD sewage treatment system. In both WWTPs, the abundance of most genes in spring and summer were higher than that of autumn and winter seasons. Taken together, changes in temperature, caused by seasonal changes, may contribute to changes in abundance of nitrogen metabolic functional genes.

## 1 Introduction

Several emerging techniques are applied in wastewater treatment plants (WWTPs) to efficiently treat different wastes. Anaerobic–anoxic–oxic (A^2^/O) and deoxidation ditch (DOD) processes have increasingly been preferred because of their efficiency in treating various wastewater within WWTPs (Das and Singh, 2022). A^2^/O, a wastewater treatment process, involves a sequence of treatment zones designed to eliminate organic matter and nutrients of nitrogen and phosphorus from wastewater (Moharram et al., 2017). The A^2^/O process is a cyclic operation, with the wastewater flowing sequentially through anaerobic, anoxic, and oxic zones. This configuration allows the efficient removal of organic matter, nitrogen, and phosphorus, making it an effective treatment method for wastewater before its discharge into receiving bodies of water or reuse in various applications (Gizińska-Górna and Wasąg, 2022; Moharram et al., 2017). Therefore, it is commonly used in biological nutrient removal processes to effectively reduce nitrogen and phosphorus concentrations in wastewater. DOD, another wastewater treatment method, represents a specific activated sludge (AS) process to remove organic matter and nutrients from wastewater (Shammas and Wang, 2009). It is commonly employed in municipal and industrial WWTPs. In the DOD process, wastewater flows into a circular or rectangular basin, known as the ditch, equipped with mechanical or diffused aerators. The wastewater is subjected to a combination of aerobic and anoxic conditions to facilitate the treatment process (Shammas and Wang, 2009; Stamou, 1997). A^2^/O and DOD processes affect microbial ecology due to variations in their operating conditions, treatment mechanisms, and the types of microorganisms involved (Feng et al., 2022; Park et al., 2021). Both processes have demonstrated efficiency in nitrogen removal, but their performance can vary significantly due to seasonal and geographic factors, which influence microbial activity and gene expression (Gizińska-Górna and Wasąg, 2022; Park et al., 2021; Shammas and Wang, 2009).

Seasonality can have a significant impact on WWTP performance. Moreover, seasonality can influence WWTP performance by affecting flow rates, organic load, nutrient balance, temperature conditions, algal blooms, and sludge handling (Thobejane et al., 2023). Understanding and adapting to these seasonal variations are crucial for optimizing treatment processes, ensuring compliance with regulations, and maintaining the overall efficiency and effectiveness of WWTP operations (Makuwa et al., 2022). Cold temperatures can significantly impact WWTP efficiency and performance. The performance and microbial conditions of a WWTP during autumn or winter can equally be influenced by various factors, including the characteristics of the influent wastewater, weather conditions, and seasonal variations in pollutant loadings (Dai et al., 2023; Guerrero et al., 2011). The performance and microbial conditions of WWTPs depend on various factors, including specific characteristics of the influent wastewater, the design and capacity of the treatment plant, and any seasonal variations in pollutant loadings (Bhatt et al., 2020; Bwapwa, 2022). These variations can be driven by regional and local factors, including climate, geographic location, and specific seasonal patterns. Monitoring and adapting treatment processes based on influent characteristics, flow rates, and other seasonal variations are essential to maintain optimal performance and meet regulatory standards (Guerrero et al., 2011; Liu et al., 2016).

Recent studies have emphasized the importance of nitrogen metabolism functional genes, including *amoA* (ammonia-oxidizing bacteria), *nirS* and *nirK* (denitrification genes), and *nosZ* (nitrous oxide reductase), in the biological nitrogen removal process (Arumugham et al., 2024; Galloway et al., 2008; Zheng et al., 2024). These genes regulate the nitrification and denitrification stages, which are sensitive to environmental factors like temperature and dissolved oxygen (DO) levels (Li et al., 2018; Potgieter et al., 2020; Zhang et al., 2019). Seasonal variations in nitrogen functional genes and nitrogen metabolism in WWTPs can occur due to changes in influent characteristics, temperature, hydraulic loading, and microbial community dynamics (Liu et al., 2016). These variations can be noticeable in nitrification and denitrification genes, nitrogen metabolism, temperature effects, organic loadings, and nutrient levels (Li et al., 2015). Specific dynamics of nitrogen functional genes and nitrogen metabolism depend on the design and operation of WWTPs, regional climate, and the specific characteristics of the influent wastewater. Regular monitoring of nitrogen-related parameters, including gene expression, nutrient concentrations, and treatment performance, can provide insights into seasonal variations and guide operational adjustments to optimize nitrogen removal efficiency throughout the year (Li et al., 2015; Zhang et al., 2019).

Despite the known influence of seasonality, few studies have conducted comprehensive comparisons of microbial dynamics and functional gene abundance across different WWTP processes in distinct climatic regions. Previous research has primarily focused on how operational parameters such as hydraulic retention time and sludge age affect nitrogen removal, but the response of A^2^/O and DOD systems to geographical and seasonal variations remains less explored (Song et al., 2021; Thobejane et al., 2023). Moreover, understanding how these factors impact the abundance of specific nitrogen metabolism functional genes can provide deeper insights into microbial activity and system performance under varying conditions.

Therefore, this study aimed to evaluate the differences in the abundance of nitrogen removal functional microbes based on high-throughput experimental results between different sewage treatment processes within A^2^/O and DOD processes in the humid Wuhan and semiarid Xi’an cities, China. The study focused on analyzing the process function differences and comparing and analyzing the functional genes of nitrogen removal microorganisms in different seasons of the two sewage treatment processes through quantitative polymerase chain reaction (qPCR) technology. The overall goal was to compare the effects of the treatment process and environmental conditions such as geographic and seasonal variations on the nitrogen removal performance of sewage treatment plants and provide the theoretical basis and implications for optimizing nitrogen removal-related processes. It was hypothesized that there is no variation in the levels of functional genes and nitrogen metabolism between treatment systems and across different seasons.

## 2 Materials and methods

### 2.1 Experimental design and sampling

This study was undertaken in two WWTPs marked as B and T. WWTP B in Xi’an City primarily accepts and treats production wastewater from industrial enterprises and domestic sewage from residential areas in the surrounding areas of the plant. It has a wastewater mixing ratio of ~3:7, a service area of 85 km^2^, a service population of 1.2 million people, and daily treatment volume of ~150,000 tons. The daily treatment capacity of DOD is ~100,000 tons and that of modified A^2^/O is ~50,000 tons; the sewage treatment follows the national level-A effluent standard. The main pollutants in the influent water of this WWTP are biological oxygen demand (BOD), suspended solids, chemical oxygen demand (COD), ammonia–nitrogen (NH_4_^+^–N), and other pollutants. The main industrial wastewater discharges in the basin are primarily electronics, pharmaceuticals, leather, coking, paper, etc. WWTP T in Wuhan primarily accepts and treats domestic wastewater and possible partial production wastewater from residents in the surrounding areas of the plant, with a service area of 43 km^2^, a service population of 330,000 people, and a small number of biopharmaceutical enterprises and electronic product processing plants in the service area. The plant’s current daily treatment capacity is 100,000 tons. DOD treats 40,000 tons and A^2^/O treats 60,000 tons daily; the wastewater treatment follows the national level-A effluent standard. The main operating parameters of the two WWTPs are shown in Supplementary Table S1. The experimental design had 16 samples categories: BA1 (WWTP B with A^2^/O process sampled in spring), BA2 (WWTP B with A^2^/O process sampled in summer), BA3 (WWTP B with A^2^/O process sampled in autumn), BA4 (WWTP B with A^2^/O process sampled in winter), BD1 (WWTP B with DOD process sampled in spring), BD2 (WWTP B with DOD process sampled in summer), BD3 (WWTP B with DOD process sampled in autumn), BD4 (WWTP B with DOD process sampled in winter), TA1 (WWTP T with A^2^/O process sampled in spring), TA2 (WWTP T with A^2^/O process sampled in summer), TA3 (WWTP T with A^2^/O process sampled in autumn), TA4 (WWTP T with A^2^/O process sampled in winter), TD1 (WWTP T with DOD process sampled in spring), TD2 (WWTP T with DOD process sampled in summer), TD3 (WWTP T with DOD process sampled in autumn), and TD4 (WWTP T with DOD process sampled in winter).

Based on the knowledge that the sample of the aeration tank in the biochemical section can wholly represent the entire sewage treatment plant, the slurry mixed with water at the outlet of the aeration tank was selected as the representative sample. To achieve that, a mixture of 500 mL of AS was uniformly collected in triplicate in a sterile sampling bottle. The influent sample from each sampling point was also collected similarly for water quality determination.

### 2.2 Measurement of physicochemical parameters

The collected AS and sewage samples were immediately transferred to the laboratory in an ice box and temporarily stored at 4°C. AS samples were centrifuged at 10,000 × *g* at 4°C for 15 min and frozen at −80°C for DNA extraction. The water quality indexes of the influent water samples were determined by national standards: total nitrogen (TN) was determined by alkaline potassium persulfate digestion ultraviolet spectrophotometry (HJ 636–2012), NH_4_^+^–N was determined by Knotler reagent spectrophotometry (HJ 535–2009), total phosphorus (TP) was determined by ammonium molybdate spectrophotometry (GB 11893–89), BOD_5_ was determined by dilution and inoculation (GB 7488–87), and COD was determined by rapid digestion spectrophotometry (HJ/T 399–2007).

### 2.3 Sample DNA extraction

For DNA extraction, 0.9 g of the collected AS sample was weighed, DNA was extracted using a DNA Isolation Kit (E.Z.N.A., Omega, Norcross, GA, United States) and DNA concentration and purity were determined. The entire procedure was performed according to the manufacturer’s instructions. The obtained DNA solution was analyzed by 1% agarose electrophoresis, and its concentration and purity were verified by a Nanodrop microspectrophotometer.

### 2.4 High-throughput sequencing and data processing

This experiment utilized the Ion Torrent IonS5™ XL high-throughput sequencing technology. The bacterial 16S rDNA V4 region of DNA samples which passed quality control was amplified using barcoded specific primers 515F/806R and Phusion^®^ High-Fidelity PCR Master Mix with GC Buffer from New England Biolabs (Li et al., 2024). Sequencing data underwent initial quality control using Cutadapt version 1.9.1 to obtain raw reads, followed by chimera removal using Vsearch to yield clean reads (Rognes et al., 2016). All sequencing data for microbial communities of municipal WWTPs were submitted to the National Center of Biotechnology Information (NCBI) Sequence Read Archive under accession numbers PRJNA605862 and PRJNA606090.

During data processing, clean data were clustered into Operational Taxonomic Units (OTUs) with 97% identity using Uparse version 7.0 (Haas et al., 2011). OTU sequences were annotated using Mothur version 1.40 and the SILVA132 SSU rRNA database with a threshold of 0.8 ~ 1 to obtain taxonomic information and to analyze community composition at various taxonomic levels (Edgar, 2013). Multiple sequence alignment of all OTU sequences was performed using MUSCLE version 3.8 to establish their phylogenetic relationships (Quast et al., 2012).

### 2.5 Primer selection for denitrification-related genes

To study the difference in nitrogen removal performance between different samples, qPCR was performed for the relevant functional genes in the main nitrogen removal process. Ammonia-oxidizing bacteria (AOB) 16S rRNA genes, which cover a wider area, and *amoA*, which provides more functionally relevant information about nitrifying bacterial populations, were selected as primers for AOB studies. Owing to the sequence diversity of nitrite-oxidizing bacteria (NOB) and to fully reflect the abundance of the taxa in the sample, multiple primer pairs were used to analyze the characteristics of the taxa. These included *nxrA* and *nxrB* for *Nitrobacter* and *nxrB* for *Nitrospira* to distinguish specific nitrite-oxidizing microbes. For the comammox study, although relevant or combination primers were used to study the ecological abundance, their effect in pre-experiment was stable due to the source of primers or samples, so this part of the experiment was omitted. For denitrifying bacteria, *nirS* and *nirK* genes, with the same function but within a different niche, were selected to detect the abundance of nitrite-reducing bacteria. Primers targeting the *nosZ* gene were selected to measure the ability to perform complete nitrification and denitrification. The anammox 16S rRNA gene was selected as a primer to determine the abundance of anaerobic AOB. Information and references related to primers are detailed in Table 1.

**Table 1:** Primers suitable for amplifying 16S rRNA and functional genes of denitrification.

Target group	Target gene	Primer name	Primer sequence (5′-3′)	qPCR program (40 cycles)	Reference
Bacteria	16S rRNA	338F	ACTCCTACGGGAGGCAGCAG	5 min at 95°C, 10 s at 95°C, 20 s at 57°C, 30 s at 72°C	Muyzer et al. (1993)
		518R	ATTACCGCGGCTGCTGG		
Betaproteobacterial AOB	AOB 16S rRNA	CTO189f*	GGAGAAAAGCAGGGGATCGGGAGGAAAGCAGGGGATCGGGAGGAAAGTAGGGGATCG	5 min at 95°C, 10 s at 95°C, 20 s at 57°C, 30 s at 72°C	Chakravorty et al. (2007)
		CTO654r	CTAGCYTTGTAGTTTCAAACGC		
Betaproteobacterial AOB	*amoA*	AmoA-1F	GGGGTTTCTACTGGTGGT	5 min at 95°C, 10 s at 95°C, 20 s at 60°C, 30 s at 72°C	Rotthauwe et al. (1997)
		AmoA-2R	CCCCTCKGSAAAGCCTTCTTC		
*Nitrobacter*	*nxrA*	F1nxrA	CAGACCGACGTGTGCGAAAG	5 min at 95°C, 10 s at 95°C, 20 s at 55°C, 30 s at 72°C	Rani et al. (2017)
		R2nxrA	TCCACAAGGAACGGAAGGTC		
*Nitrobacter*	*nxrB*	nxrB1F	ACGTGGAGACCAAGCCGGG	5 min at 95°C, 10 s at 95°C, 20 s at 55°C, 30 s at 72°C	Pester et al. (2014)
		nxrB1R	CCGTGCTGTTGAYCTCGTTGA		
*Nitrospira*	*nxrB*	nxrB169f	TACATGTGGTGGAACA	5 min at 95°C, 10 s at 95°C, 20 s at 57°C, 30 s at 72°C	Pester et al. (2014)
		nxrB638r	CGGTTCTGGTCRATCA		
Denitrifying bacteria	*nirK*	nirK876F	ATYGGCGGVCAYGGCGA	5 min at 95°C, 10 s at 95°C, 20 s at 57°C, 30 s at 72°C	Throbäck et al. (2004)
		nirK1040R	GCCTCGATCAGRTTRTGGTT		
Denitrifying bacteria	*nirS*	nirScd3aF	GTSAACGTSAAGGARACSGG	5 min at 95°C, 10 s at 95°C, 20 s at 57°C, 30 s at 72°C	Throbäck et al. (2004)
		nirSR3cdR	GASTTCGGRTGSGTCTTGA		
Denitrifying bacteria	*nosZ*	nosZ1F	WCSYTGTTCMTCGACAGCCAG	5 min at 95°C, 10 s at 95°C, 20 s at 60°C, 30 s at 72°C	Throbäck et al. (2004)
		nosZ1R	ATGTCGATCARCTGVKCRTTYTC		
Anammox bacteria	16S rRNA	AMX809F	GCCGTAAACGATGGGCACT	5 min at 95°C, 10 s at 95°C, 20 s at 60°C, 30 s at 72°C	Yang et al. (2020)
		AMX1066R	AACGTCTCACGACACGAGCTG		

### 2.6 Gene cloning and plasmid construction and extraction

Using primers described in Table 1, the DNA sample in Supplementary Table S1 was selected as the template, and PCR amplification of functional genes was performed using PCR 2× Es Taq Master Mix (Kangwei CW0690L, China). The PCR procedure was described previously (Braker et al., 1998; Geets et al., 2007). The PCR products were detected by 1% agarose electrophoresis, fragment size was compared to those existing in the literature, and the gel recovered after detection was confirmed to be accurate. The Agarose Gel DNA Recovery Kit (Kangwei CW2302S, China) was used according to the manufacturer’s instructions.

For the plasmid construction experiment, the pMD^®^ 18-T Vector Cloning Kit (TaKaRa, Dalian, China) was used for T-carrier ligation and transferred into DH5α-competent cells with a plasmid size of 2,692 bp. The experimental steps included T vector ligation, heat shock conversion, culture, and identification. After incubating the correct clone in LA liquid bares overnight, the plasmid was extracted using the Fast Plasmid DNA Small Quantity Kit (Simgen, Hangzhou, China) according to the manufacturer’s instructions. The plasmids used in the experiment are listed in Supplementary Table S2.

### 2.7 qPCR of samples and establishment of their standard curves

For the qPCR of samples and establishment of their standard curves, SYBR Green I dye with Hieff^®^ qPCR SYBR^®^ Green Master Mix (No Rox; Yisheng, Shanghai) was used as the reagent. The Light Cycler 480 instrument (Roche, Switzerland) was used for this process (Yang et al., 2020).

The standard plasmid copy number was calculated by applying the plasmid concentration value to Equations 1, 2.


(1) Plasmid size=Tvector size+target gene size

(2) $$\begin{array}{l} \mathrm{Copy number}\;\left(\mathrm{copies}/\mathrm{\mu L}\right)=6.02\times 10^{23}\times \\ \mathrm{plasmid concentration}/\left(660\times \mathrm{plasmid size}\right) \end{array}$$

The obtained plasmid stock solution was diluted to 10^−8^ by a 10-fold concentration gradient, the reaction system was prepared using each concentration gradient as a template, and the corresponding annealing temperature was set for qPCR. The *C*t value corresponding to each measured concentration was recorded, the *C*t value was denoted on the *X*-axis and the log (copies) value was denoted on the *Y*-axis to establish a standard curve.

The extracted DNA was used as the template for qPCR, with three parallel experiments per sample, and the reaction system was the same as mentioned in Table 1. The *C*t value measured by each sample was marked into the corresponding standard curve to calculate the copy number and corresponding copy number concentration value.

### 2.8 Statistical analysis

For data analysis and visualization, Qiime version 1.9 software (Caporaso et al., 2010) was used to calculate diversity indices such as Observed-otus, Chao1, Shannon, Simpson, Ace, and Goods-coverage. *α*- and *β*-Diversity indices and group differences were analyzed using R version 2.15 software. Qiime was used to calculate Unifrac distances and construct an unweighted pair group method with arithmetic mean sample cluster trees. R version 22.15, along with its vegan, weighted gene co-expression network analysis, stat, FactoMineR, and ggplot2 packages, was used to plot rarefaction curves, rank abundance curves, species accumulation curves, principal component analysis (PCA), principal coordinates analysis (PCoA), and non-metric multidimensional scaling plots, and conduct canonical correspondence analysis, redundancy analysis, and environmental factor correlation analysis (Gupta et al., 2018).

Independent- and paired-sample *t*-tests were performed to analyze the process (A and D) and sewage treatment plant (B and T), respectively, and to compare the differences in functional genes between different processes and sewage treatment plants. One-way analysis of variance (ANOVA) was used for differences between seasons; two-way ANOVA was used for interaction between processes, WWTPs, and seasons; and the Student’s–Newman–Keuls’ method was used for multiple comparisons. The above analysis was done using SPSS version 23.0 (IBM, United States). The relationship between the physical and chemical indexes of influent water and the abundance of different denitrification functional genes was analyzed by CANOCO 5.0 for RDA. PAST version 4.0 was used for the PCA of individual gene abundance differences between samples.

## 3 Results

### 3.1 WWTP operation and performances

The physicochemical parameters across different WWTPs during different seasons are provided in Supplementary Table S1 and Supplementary Figure S1. Statistics results showed that there were significant differences (*p* < 0.05) in BOD_5_, TP, and SV_30_ between T and B, but there was no difference in temperature, pH, COD, TN, and NH_4_^+^–N, and no significant difference in all parameters in different seasons. On average, temperature and pH values were higher in WWTP T than those in WWTP B. However, COD, BOD_5,_ TP, TN, NH_4_^+^–N, and SV_30_ values were lower in WWTP T than those in WWTP B. SV_30_ values were generally lower in the A^2^/O system than those in the DOD sewer system. The concentration of each pollutant in the influent of WWTP B was significantly higher than that of WWTP T, which may be due to the presence of industrial effluent in the influent.

While ensuring the operation and normal effluent quality of WWTPs, SV_30_ values, which reflect sludge settling performance, compared across various processes of the same WWTP revealed that the sludge settling performance between the two processes was partially different, possibly because of the maintenance of different sludge concentrations and sludge return ratios. Furthermore, the SV_30_ value of WWTP B was relatively high, especially in winter, indicating that the sludge settling performance of WWTP B was poor during the winter, with sludge bulking even occurring.

### 3.2 Sequencing statistics and microbial diversity

#### 3.2.1 Sequencing data

The quality control procedure, obtained 584,822 sequences and 130,189 OTUs were obtained via high-throughput sequencing. The rarefaction curve (Supplementary Figure S2) demonstrates that the curve levels off when the sequencing depth reaches 40,000 tags, indicating that the present sequencing data is sufficient and reasonable (Lundberg et al., 2013).

**Figure 1:**
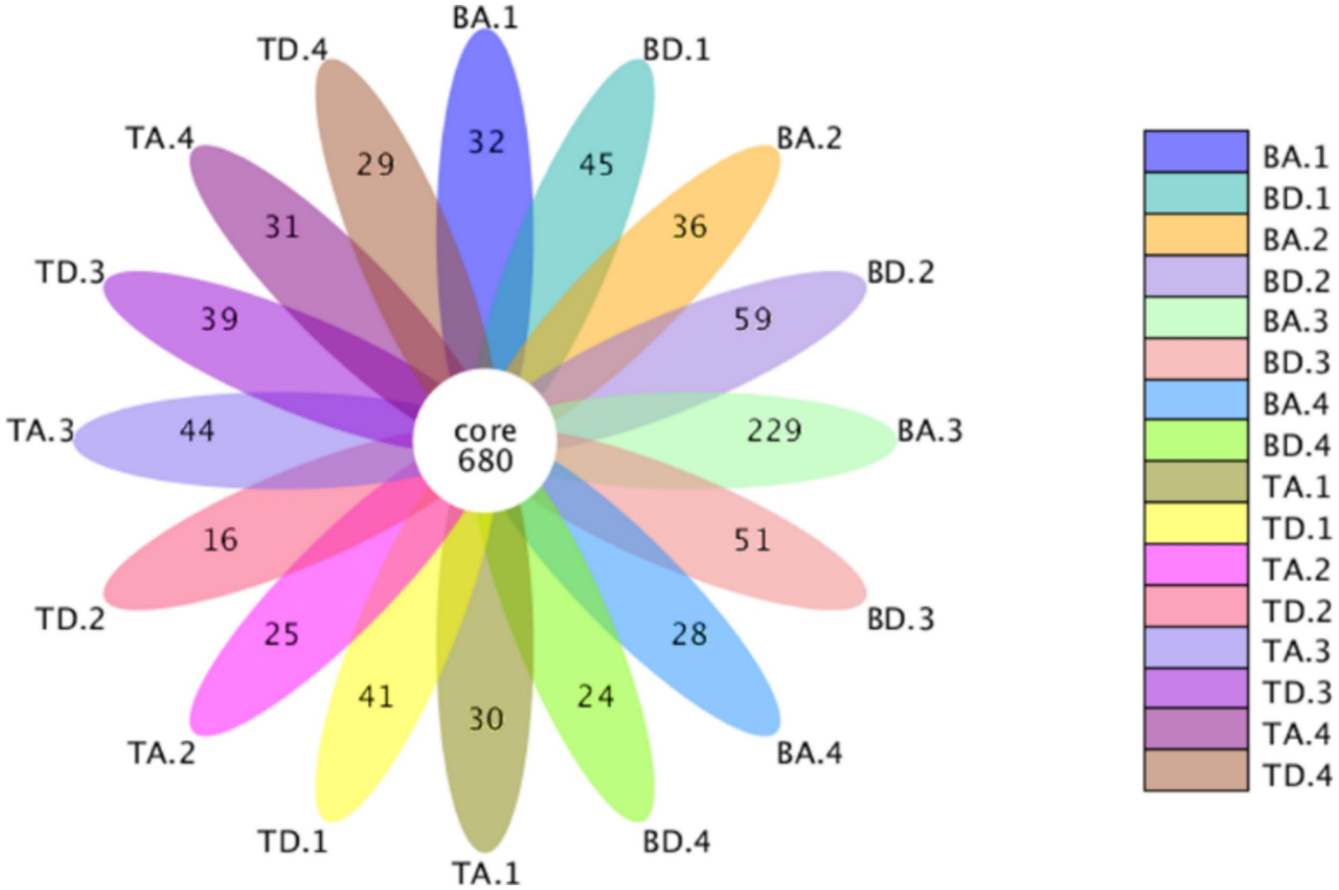
Flower diagram showing the shared and unique OTUs in groups.

Operational Taxonomic Units analysis in the different groups. When samples were grouped according to different processes and seasons between the two WWTPs, 680 core OTUs were shared among all groups (Figure 1), 1,228 core OTUs were shared among groups at WWTP B (Supplementary Figure S3), and 835 core OTUs among groups at WWTP T (Supplementary Figure S3), indicating that WWTP B possessed a larger core microbial community. Under consistent sequencing depth conditions, OTU values at WWTP B were slightly higher than those at WWTP T (Supplementary Table S3), suggesting that WWTP B may have a higher community diversity than WWTP T.

#### 3.2.2 *α*-diversity

**Figure 2:**
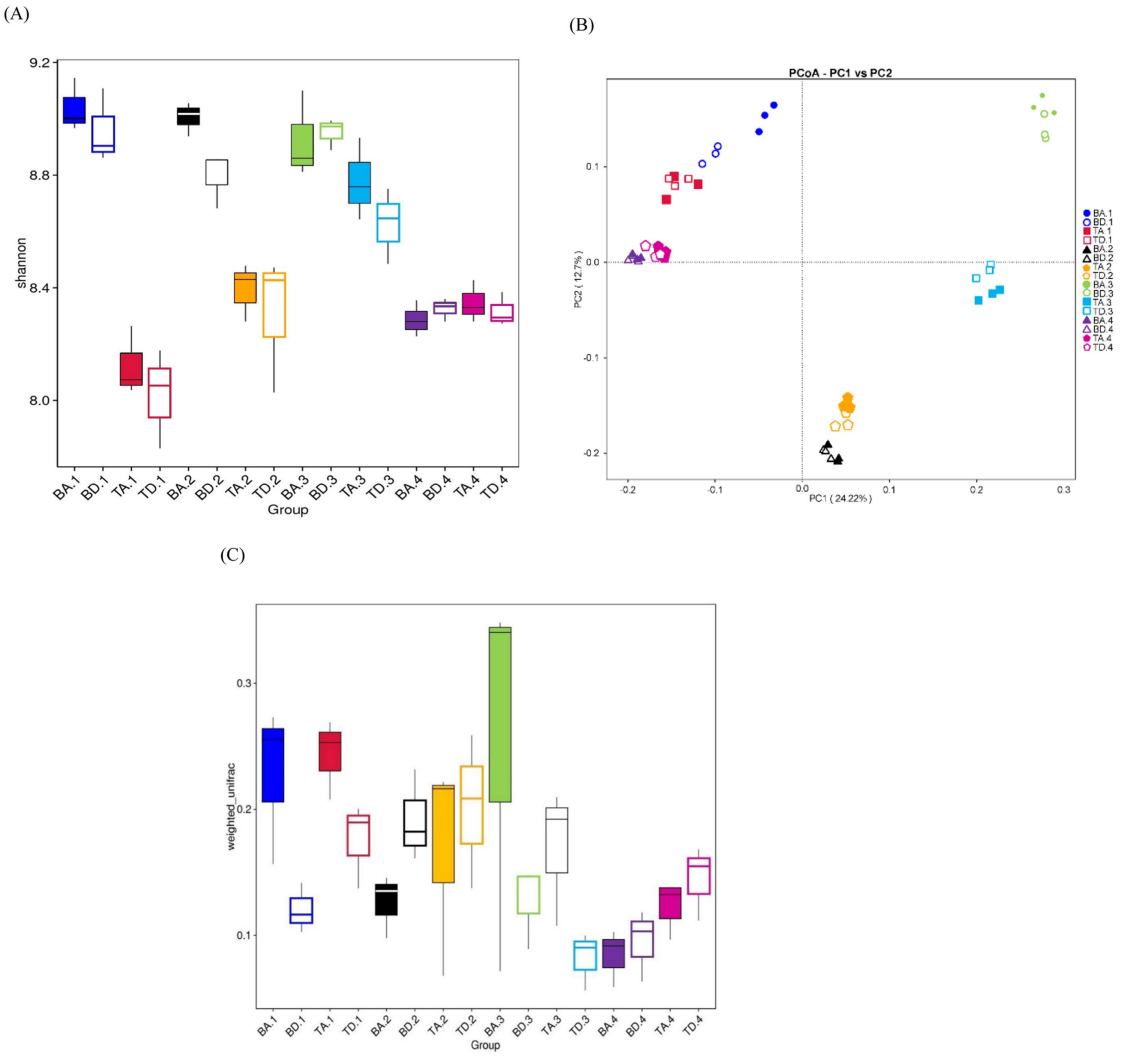
*α*- and *β*-diversity among all groups. (A) Boxplots for *α*-diversity of Shannon-Wilcox indices; same color indicates the same season and location, and the same color with a different shape indicates a different process. (B) PCoA plot based on unweighted_unifrac distances of each group. (C) Boxplots for *β*-diversity indices based on unweighted_unifrac distances of each group.

The results on *α*-diversity across groups are presented in Supplementary Table S3, with the boxplot of the Shannon Index for different groups shown in Figure 2A. Comparing the different processes, the *α*-diversity of the A^2^/O system was partially higher and more stable than that of DOD processes across all seasons. Although there were some variations in Shannon Indices, these differences were not statistically significant, except for a significant difference observed in summer (Wilcoxon rank-sum test, *p* < 0.05), suggesting that the more complex habitat of the A^2^/O process supports a more diverse microbial community structure.

On comparing different sites, the *α*-diversity of WWTP B was significantly higher than WWTP T in all seasons except winter, in which the difference between the two WWTPs was insignificant (Wilcoxon rank-sum test, *p* < 0.05). This could be attributed to WWTP B receiving a certain amount of industrial wastewater with a higher pollutant load, leading to a more complex microbial community in AS.

#### 3.2.3 *β*-diversity

The PCoA plot shows that samples from the same city and season consistently cluster together in proximity, indicating that the *β*-diversity difference between the A^2^/O and DOD processes is insignificant. However, the distance between samples from different seasons within the same city is greater than that between samples from different locations in the same season, suggesting a seasonal variation in *β*-diversity, with differences between seasons being more pronounced than those between processes and locations (Figure 2B).

The *β*-diversity of A^2^/O processes is generally higher than that of DOD processes in spring and autumn, but lower in summer and winter. This suggests that the differences in *β*-diversity between the A^2^/O and DOD processes are influenced by seasonal factors, possibly because of the presence of temperature-sensitive taxa in A^2^/O processes (Figure 2C).

#### 3.2.4 Microbial community analysis

The taxonomic annotation results of OTUs at the phylum level revealed that the predominant phyla across all samples included *Proteobacteria*, *Bacteroidetes*, *and Chloroflexi*, collectively accounting for 68% of the total abundance. Other phyla, such as *Nitrospirae*, *Actinobacteria*, *Planctomycetes*, *Firmicutes*, and *Acidobacteria*, each accounted for >1% of the total abundance (Supplementary Figure S4). Within these major phyla, the predominant class level included *Alphaproteobacteria*, *Gammaproteobacteria*, and *Deltaproteobacteria* within *Proteobacteria*, *Bacteroidia* within the Phylum *Bacteroidetes*; and *Anaerolineae* along with *unidentified_Chloroflexi* within the *Chloroflexi*. The composition of these phyla is consistent with previous reports on the microbial community structure of AS. The seasonal fluctuations in the abundance of these major microbial groups were more pronounced in WWTP B than in WWTP T. Among the dominant phyla with abundances >1%, *Nitrospirae*, *Planctomycetes*, and *Firmicutes* showed significant differences between the A^2^/O and DOD processes. *Nitrospirae* and *Planctomycetes* were more abundant in the DOD processes, whereas *Firmicutes* had higher abundances in the A^2^/O processes (Supplementary Figure S4).

**Figure 3:**
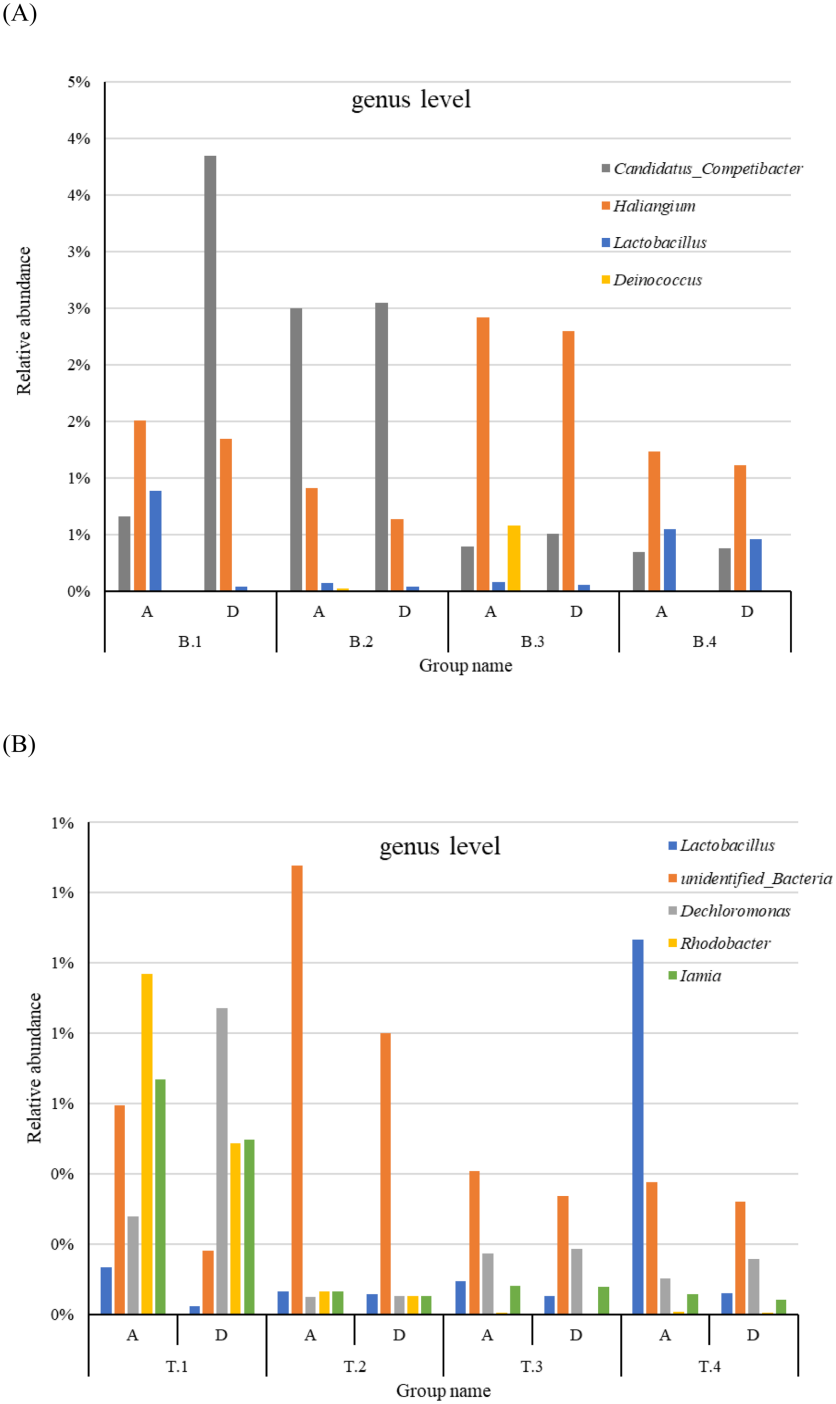
Relative abundance at the genus level showing differences between the A^2^/O and DOD processes in WWTP B (A) and WWTP T (B). T and B Representing Wuhan and Xi’an respectively; A and D represents A^2^/O and DOD processes respectively; 1, 2, 3, and 4, respectively, represent the four seasons of spring, summer, autumn and winter.

Although significant variations were observed among subgroups at the genus level, the genera *Dokdonella*, *unidentified_Nitrospiraceae*, *Terrimonas*, and *unidentified_Chloroflexi* were present in all sample groups with abundances exceeding 0.5%. The abundances of *Terrimonas*, *unidentified_Nitrospiraceae*, *and Rhodococcus*, which were reported as dominant genera (Zhang et al., 2019), varied notably, as shown in Supplementary Figure S4. This indicates that while the abundance of microbial communities in AS significantly fluctuates in the genus level because of environmental influences, the core community structure remains relatively stable. Significant differences in abundance and composition were observed across groups from different locations and seasons (Supplementary Figure S4). In WWTP B, the microbial community structure remained relatively stable during spring, summer, and autumn, however, the abundance of *unidentified_Nitrospiraceae* and *Dokdonella* increased substantially in winter. In contrast, in WWTP T, *Terrimonas*, *unidentified_Nitrospiraceae*, *and Dokdonella* were more abundant in spring, with their proportions gradually decreasing through summer, autumn, and winter, whereas the abundance of *unidentified_Flavobacteriales* and *Candidatus_Competibacter* steadily increased across all four seasons. Among the top 30 dominant genera, *Candidatus Competibacter* was more abundant in DOD processes, whereas *Haliangium*, *Lactobacillus*, and *Deinococcus* were more prevalent in the A^2^/O process in WWTP B (Figure 3A). In WWTP T, *Lactobacillus*, *unidentified Bacteria*, *Rhodobacter*, and *Iamia* had higher abundance in the A^2^/O process, whereas *Dechloromonas* was more abundant in DOD processes (Figure 3B).

### 3.3 Abundance of total bacterial 16S rRNA genes and nitrogen metabolism functional genes

This study employed a comparative approach to investigate the differences in abundance of functional genes involved in nitrogen metabolism, including in *16S rRNA*, *AOB-16S rRNA*, *AOB-amoA*, *Nitrobacter nxrA*, *Nitrobacter nxrB*, *Nitrospira nxrB*, *nirS*, *nirK*, *nosZ*, *and anammox 16S rRNA*. The results are presented in Supplementary Table S4, which lists the copy number differences between groups for these genes.

#### 3.3.1 Total bacterial 16S rRNA gene abundance

The absolute abundance of the total bacterial 16S rRNA gene in WWTP T samples was 1.94 × 10^7^ to 4.35 × 10^7^ copies/ng, and the abundance of total bacterial 16S rRNA genes in WWTP B samples was 1.40 × 10^7^ to 6.50 × 10^7^ copies/ng, among which the fluctuation in WWTP B was more obvious, and the difference between the two processes was relatively large. OTUs results were similar, and the microbial diversity of WWTP B was slightly higher than that of WWTP T (Supplementary Figure S5).

#### 3.3.2 AOB-associated genes

**Figure 4:**
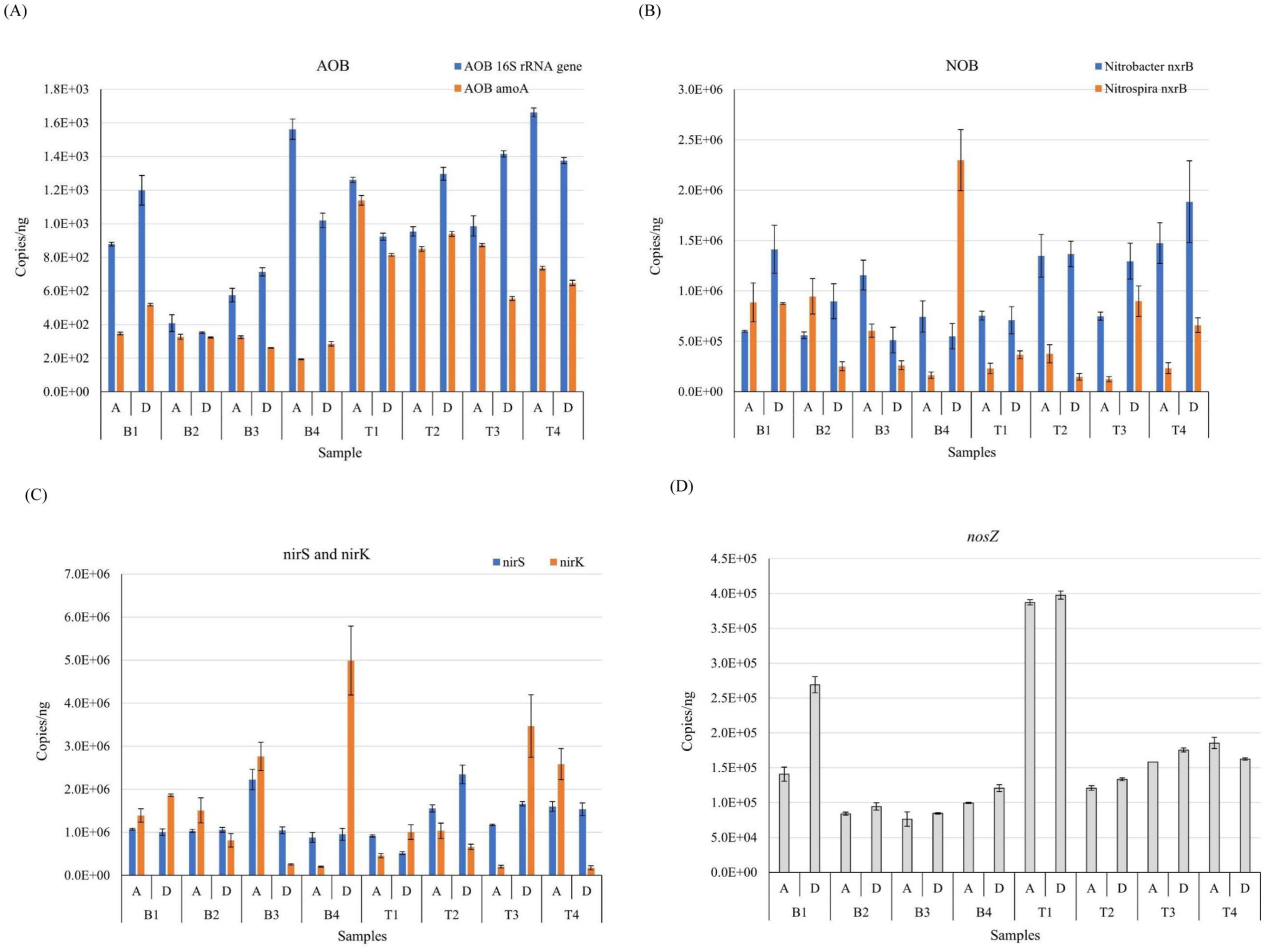
Comparison of the absolute abundance of nitrogen metabolism functional genes including AOB *16S rRNA* gene and AOB *amoA* gene (A), *Nitrobacter nxrB* and *Nitrospira nxrB* gene (B), *nirS* and *nirK* gene (C), and *nosZ* gene (D) by qPCR (copies/ng DNA) in different seasons and processes of WWTPs B and T. T and B represent Wuhan and Xi’an respectively; A and D represent A^2^/O and DOD processes respectively; 1, 2, 3, and 4 represent the four seasons of spring, summer, autumn and winter, respectively.

The absolute abundance of AOB *amoA* and *16S rRNA* genes in the samples was detected in both WWTPs. The abundance of AOB *amoA* and *16S rRNA* genes in WWTP B was 1.94 × 10^2^ to 5.18 × 10^2^ and 3.53 × 10^2^, respectively, whereas the abundance of AOB *amoA* and *16S rRNA* genes in WWTP T samples was 5.55 × 10^2^ to 1.14 × 10^3^ and 9.23 × 10^2^ to 1.66 × 10^3^, respectively (Figure 4A). Due to the coverage of different primers, the AOB *16S rRNA* gene had more copies than AOB *amoA* (Abellan-Schneyder et al., 2021). Comparative analysis between Wuhan and Xi’an revealed significant differences in the absolute abundance of AOB *amoA* and *16S rRNA* genes between WWTPs T and B (*p* < 0.05). Overall, the absolute abundance was higher in WWTP T than in WWTP B. When comparing the copy number of the two genes relative to the bacterial *16S rRNA* gene, only AOB *amoA* showed a significant difference between the two locations. Although the NH_4_^+^–N concentration in the influent water of WWTP B was generally higher than that in WWTP T, the AOB *amoA* abundance of each group of samples from WWTP B was lower than that of WWTP T.

Seasonal comparative analysis indicated that the absolute abundance of AOB *amoA* varied significantly across seasons (*p* < 0.05), although there was no significant difference in the AOB *16S rRNA* gene. The abundance of the AOB *16S rRNA* gene and *amoA* in summer and autumn was relatively consistent, but there were substantial differences between spring and winter, particularly in WWTP B. This variation may be attributed to the greater impact of seasonal temperature differences on AOB *amoA*. Comparative analysis of different processes showed that the process type did not significantly affect the abundance of AOB *16S rRNA* and *amoA* genes. There was no significant correlation between the interactions of the two gene abundances across different processes, locations, and seasons.

#### 3.3.3 NOB-associated genes

The absolute abundance of *Nitrobacter nxrA* and *nxrB* and *Nitrospira nxrB* in WWTP B samples were 8.62 × 10^1^ to 2.99 × 10^2^, 5.12 × 10^5^ to 1.41 × 10^6^, and 1.63 × 10^5^ to 2.30 × 10^6^, respectively. In contrast, the abundance of *Nitrobacter nxrA* and *nxrB* and *Nitrospira nxrB* in samples from WTTP T was 1.15 × 10^2^ to 1.83 × 10^2^, 7.10 × 10^5^ to 1.89 × 10^6^, and 1.23 × 10^5^ to 8.99 × 10^5^, respectively. Because the copy number of *Nitrobacter nxrA* differed from that of *Nitrobacter nxrB* and *Nitrospira nxrB* by nearly three orders of magnitude in samples (Figure 4B; Supplementary Figure S5). The absolute abundance of *Nitrobacter nxrB* was higher than that of *Nitrospira nxrB* in most groups, and the difference between them was smaller in WWTP B than in WWTP T. This may be due to differences in influent water quality or operational conditions, resulting in a higher abundance of the R-countermeasure *Nitrobacter* better adapted to the environment of high nitrite and high oxygen concentrations than the K-countermeasure *Nitrospira* which was better adapted to the environment of low nitrite and low oxygen concentrations (Blackburne et al., 2007; Dytczak et al., 2008; Kim and Kim, 2006). The absolute abundance of *Nitrobacter nxrA* did not differ significantly between processes, locations, seasons, and interactions with each other.

#### 3.3.4 Denitrification-related genes

The absolute abundance of *nirS*, *nirK*, and *nosZ* genes in the samples revealed copy number ranges of 8.80 × 10^5^ to 2.22 × 10^6^, 2.03 × 10^5^ to 4.99 × 10^6^, and 7.63 × 10^4^ to 2.69 × 10^5^ in WWTB B and 5.18 × 10^5^ to 2.35 × 10^6^, 1.75 × 10^5^ to 3.47 × 10^6^, and 1.21 × 10^5^ to 3.98 × 10^5^, respectively, in WWTP T (Figures 4C,D). Results on the differences in copy number of *nirS*, *nirK*, and *nosZ* in different processes, locations, and seasons showed that the absolute abundance of *nirS* and *nirK* was insignificant between different seasons and locations, but there was a significant difference in the copy number of *nosZ* between seasons and locations (*p* < 0.05), and multiple comparisons showed that the difference between the first and other quarters was the most pronounced among seasonal differences. Among different processes in all other subgroups, except for the fourth quarter of WWTP T, the absolute abundance of *nosZ* in DOD was higher than in A^2^/O. The highest and average values of absolute *nosZ* abundance were significantly higher in WWTP T than those in WWTP B at different sites. Although *nirS* and *nirK* in different ecological niches had the same function, they were unrelated. In the denitrification process, WWTP T produced less N_2_O than WWTP B.

#### 3.3.5 Anaerobic ammonia oxidation

Results on the absolute abundance for anammox *16S rRNA* gene in the samples showed that WWTP B had a copy number of 4.66 × 10^4^ to 5.12 × 10^5^, whereas the WWTP T copy number was 6.46 × 10^4^ to 4.43 × 10^5^ (Supplementary Figure S5). Comparative analysis of the anammox *16S rRNA* gene showed that differences were not significant across processes, locations, seasons, and interactions, and seasonal differences were random, possibly due to the sensitivity of functional taxa to dissolved oxygen (DO).

### 3.4 Effects of influent water quality on denitrification gene abundance

**Figure 5:**
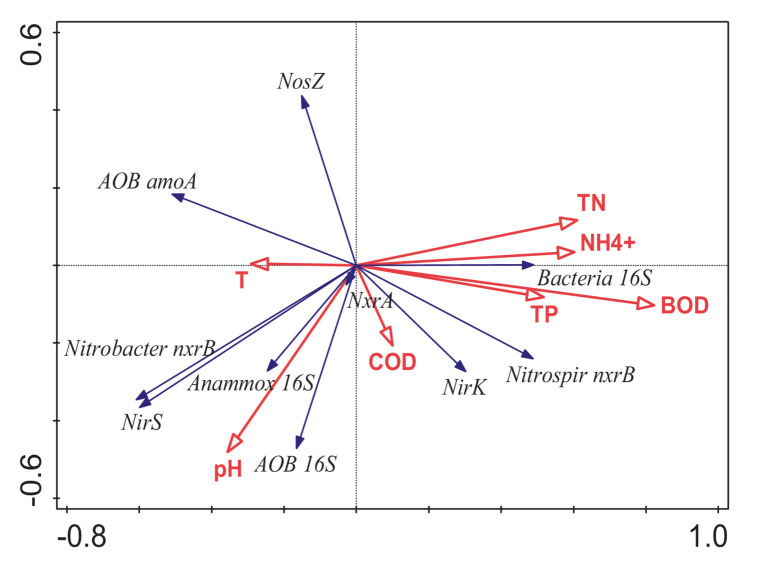
RDA of the relationship between the influent environmental variables and the absolute abundance of genes; arrow length represents the strength of the correlation between the environmental variables and the absolute abundance of genes; longer the arrow length, the stronger the correlation. The perpendicular distance between the influent environmental variables and absolute abundance of genes axes in the plot reflects their correlations. The smaller the distance, the stronger the correlation. BOD, biochemical oxygen demand for 5 days; pH, pH value; NH_4_^+^–N, ammonia nitrogen concentration; TN, total nitrogen concentration; COD, chemical oxygen demand concentration; DO, dissolved oxygen; T, temperature.

The results of the RDA analysis of the abundance of each gene and physicochemical properties of the influent water are shown in Figure 5. The abundance of the total bacterial *16S rRNA* gene was positively correlated with BOD, NH_4_^+^–N, TP, and TN; that is, the total bacterial growth was promoted when there was nutrient-rich status. *nirS* had a strong positive correlation with pH during the denitrification process, and a positive correlation between *nirK* and nutrient sources was observed. In the final step of denitrification, the abundance of *nosZ* was only positively correlated with temperature, comparable to the significant difference between seasons. Results showed that the significant difference in *nosZ* between seasons was primarily affected by temperature changes. Pearson correlation analysis of the relationship between the main NOB and nitrite-reducing bacteria revealed a significant positive correlation between *nirS* and the copy number of the *Nitrobacter nxrB* gene (*r* = 0.621, *p* < 0.05). Consequently, there was a significant positive correlation between *nirK* and *Nitrospira nxrB* gene copy number (*r* = 0.793; *p* < 0.01). *nirS* and *nirK* in different ecological niches may have a certain correspondence between different taxa of *nxrB*, *Nitrobacter nxrB*, and *Nitrospira nxrB*, thus, portraying mutual support.

## 4 Discussion

Differences in microbial community composition and their variations are often key to understanding the mechanisms of microbial community assembly and ecosystem functioning (Wu et al., 2019; Zhou et al., 2013). Different processes maintain distinct operational parameters, creating differentiated habitats within the biochemical reaction tanks, leading to microbial community structure variations (Park et al., 2021). Herein, the findings indicated that a comparative analysis of microbial community structures across different processes using the same influent did not reveal significant consistent differences. However, some general distinctions were observed. For instance, among the phyla with abundances >1%, *Nitrospirae* and *Planctomycetes* were more abundant in the DOD processes, whereas *Firmicutes* showed higher abundance in the A^2^/O process. Reportedly, *Nitrospirae* is the primary nitrifying phylum in AS microbial community, while *Planctomycetes* is known for anaerobic ammonia oxidation (Zheng et al., 2020). DOD processes exhibit a stronger nitrogen removal capacity compared to A^2^/O processes (Zhou et al., 2012). Therefore, the difference in the abundance of *Nitrospirae* and *Planctomycetes* may be crucial in differing nitrogen removal efficiencies observed between A^2^/O and DOD processes.

The assembly of microbiomes in WWTPs is closely related to absolute latitude, mean annual temperature, and influent COD and BOD concentrations (Song et al., 2021; Wu et al., 2019). WWTPs B and T are located in Wuhan and Xi’an, respectively, in different climatic regions of China, with distinct northern and southern climates. Variations in climate and temperature due to differing latitudes, along with differences in local dietary habits, can lead to differences in the composition of major pollutants in domestic wastewater and changes in the AS state. These factors may be the primary reasons for seasonal differences in the microbial community structure observed between the two locations. *Dokdonella* is considered a dominant genus composition in WWTPs for the antibiotic pharmaceutical industry. The elevated concentration of pharmaceutical wastewater in the influent of WWTP B during winter may explain the higher abundance of *Dokdonella* in that season. *Candidatus Competibacter* and *Flavobacterium* exhibit strong capabilities in removing COD, NH_4_^+^–N, and TN, and the NH_4_^+^–N concentration in the influent of WWTP T in the fall and winter was nearly twice as much as that in the spring and summer (Supplementary Table S1), which may be the primary reason for the increased abundance of denitrifying bacteria such as *Candidatus Competibacter* and *Flavobacterium* in autumn and winter.

Seasonality can exert a significant influence on the performance and dynamics of nitrogen functional genes within A^2^/O and DOD processes, which are commonly used in WWTPs and designed to remove nitrogen compounds through microbial processes (Li et al., 2018; Zhang et al., 2016). Nitrogen functional genes are responsible for various transformations of nitrogen within these systems (Myrold, 2021). The key genes involved in nitrogen removal include AOB genes (such as *amoA*), NOB genes (such as *nxrA*), and denitrifying bacteria genes (such as *nirS and nirK*) (Galloway et al., 2008; Liu et al., 2022). The activities and abundance of these genes are greatly influenced by environmental factors, including temperature, DO levels, and organic loading rates, which normally exhibit seasonal variations. Therefore, the influence of seasonality on nitrogen functional genes within A^2^/O and DOD systems depends on the specific system design, operational parameters, and geographical location. Furthermore, advancements in process control and optimization techniques, such as real-time monitoring and adaptive control strategies, can help mitigate the effects of seasonality and enhance the overall performance of these systems throughout the year (Holmes et al., 2019; Hu et al., 2023; Kuypers et al., 2018; Potgieter et al., 2020; Zhao et al., 2023). This study covered the contribution of seasonality on nitrogen functional genes within A^2^/O and DOD processes in Wuhan and Xi’an cities, China.

The diversity and population of AOB abundance in AS are the main factors affecting the nitrification efficiency of AS systems, and the increase in AOB diversity and population density can enhance the intensity of nitrification (Awolusi et al., 2018), indicating that WWTP B with a higher AS concentration had a higher NH_4_^+^–N removal capacity than WWTP T. AOB *amoA* could be more effective than AOB *16S rRNA* or bacterial *16S rRNA* genes, reflecting the differences in bacterial composition in different AS samples (Wang et al., 2021). Hence, AOB *amoA* is highly targeted in analyzing of ammonia oxidation functional microbes and can more accurately reflect AOB and ammonia oxidation intensity in AS. Studies have reported that NOB found in AS are primarily *Nitrobacter* and *Nitrospira*, with *Nitrospira* being relatively more dominant (Daims et al., 2015; Gonzalez-Martinez et al., 2016; Johnston et al., 2019). *Nitrobacter nxrB* was relatively dominant in most grouped samples, and the difference between the two WWTP B samples was smaller than that in the WWTP T sample. This could have been due to the difference in influent water quality of the sewage treatment plant or the operating conditions, resulting in a relatively high nitrite concentration in the biochemical pool, making it more suitable for high nitrite and oxygen concentrations. Indeed, the abundance of R-strategist *Nitrobacter* was higher than that of *Nitrospira*, adapted to low nitrite and low oxygen concentration of K-strategist (Han et al., 2017).

Nitrite oxidation is an essential step in fixed nitrogen transformation. The physiology of NOB implies that nitrite oxidation rates should be controlled by the concentration of their substrate, nitrite, and oxygen (terminal electron acceptor). Consequently, potential nitrite oxidation (PNO) is significantly positively correlated with *Nitrobacter nxrA* copy number in soil, and changes in PNO may mediate changes in the abundance ratio of *Nitrospira nxrB*/*Nitrobacter nxrA* (Attard et al., 2010; Cebron and Garnier, 2005; Han et al., 2017). Therefore, the abundance ratio can better predict the change in PNO (Florido et al., 2015). In this study, unlike the soil environment, the ratio trend of *Nitrospira nxrB* to *Nitrobacter nxrA* in the sample was almost consistent with that of *Nitrospira nxrB*, which made it difficult to comprehensively reflect the relative abundance of these two taxa of NOB, making it necessary to find suitable characterization indicators of AS microorganisms.

The changes in influent water quality often significantly affect AS microbial communities (Florido et al., 2015; Yang et al., 2013). During nitrification, the abundance of the AOB *16S rRNA* gene was positively correlated with pH, consistent with other reports (Abellan-Schneyder et al., 2021; Deissová et al., 2023). However, AOB *amoA* had a strong positive correlation with temperature, and this phenomenon was corroborated by the significant difference in AOB *amoA* copy number between seasons, indicating that the microbes encoding AOB *amoA* could be more sensitive to temperature changes, as reported previously (Lu et al., 2015; Song and Niu, 2022). Although AOB *amoA* and *16S rRNA* genes reflected the abundance of AOB, the target genes of different primers reflected different biota characteristics of the same function, that is some AOB were sensitive to pH, whereas some AOB were more sensitive to temperature.

Among NOB-related genes, *Nitrobacter nxrA* and *nxrB* and *Nitrospira nxrB*, pH and COD were the most influential on *Nitrobacter nxrA*, and all indicated a positive correlation. *Nitrobacter nxrB* had a stronger positive correlation with temperature and pH than *Nitrospira nxrB*, whereas *Nitrospira nxrB* was not sensitive to pH. Because no specific functional marker gene can target the broad functional population of NOB, the specific role of pH in influencing NOB growth remains unclear (Msimbira and Smith, 2020). Studies showed that *Nitrobacter nxrA* may positively correlate with pH and organic matter concentration (Bartosch et al., 2002; Li et al., 2019). Therefore, similar conclusions can be made in this study, although *Nitrobacter nxrA* was slightly affected by environmental factors. *Nitrospira nxrB* was more associated with NH_4_^+^–N concentration in influent water than other functional genes, indicating that *Nitrospira* plays an important role in nitrite oxidation in AS (Mehrani et al., 2020). Indeed, the absolute abundance of the anammox 16S rRNA gene during anaerobic ammonia oxidation had a strong positive correlation with pH and temperature, indicating stable anaerobic conditions and that environmental parameters are important factors affecting anammox bacterial communities that are rarely affected by nutrient source restriction (Chen et al., 2023).

As reported previously, changes in the abundance of denitrification genes *nxrB* or *nirS* were accompanied by changes in the abundance of microbes, such as *Flavobacterium* or *Candidatus Competibacter* (Braker et al., 2000; Wei et al., 2015; Yang et al., 2022). *Flavobacterium* has strong nitrogen and phosphorus removal ability (Enisoglu-Atalay et al., 2018), and *Candidatus Competibacter* plays a key role in denitrification processes (Rubio-Rincón et al., 2017). Genomic analysis of existing *Candidatus Competibacter*-related genera, which is the main nitrogen removal functional microbes in AS, through the NCBI database found a *nirS* reductase gene, indicating an association between the relative abundance of nitrogen removal microbes and the nitrogen removal functional gene. The unidentified genus of *Flavobacteriales* and *Candidatus Competibacter* may be related to the nitrogen metabolism functional genes *nirS* and *Nitrobacter nxrB*, and the main nitrogen removal functional microbes, the unidentified genus of *Nitrospiraceae*, may be related to the nitrogen metabolism functional genes *nirK* and *Nitrospira nxrB*, and these two groups could play their own roles in different ecological niches.

This study showed that the A^2^/O system exhibited higher levels of most nitrogen metabolism functional genes than those of the DOD system in WWTPs of the two locations. Spring and summer recorded higher values of most genes across all WWTPs than during autumn and winter. The finding concurred with studies that compared the A^2^/O and DOD systems in terms of nitrogen removal efficiency and abundance of nitrogen metabolism functional genes. Studies have reported higher values of key functional genes involved in nitrification (such as *amoA* for AOB) and denitrification (such as *nirS* and *nirK* for denitrifying bacteria) in the A^2^/O system compared to the DOD system (Kim and Nakhla, 2010; Xu et al., 2014). Other studies investigated the performance and microbial community composition of full-scale WWTPs employing the A^2^/O and DOD processes by analyzing the abundance of nitrogen metabolism functional genes and found that the A^2^/O system exhibited higher values for key genes involved in nitrification and denitrification compared to the DOD process (Ding et al., 2006; Wang et al., 2019; Xu et al., 2014). Other studies compared A^2^/O system to the sequencing batch reactor (SBR) process in a full-scale WWTP. It evaluated the nitrogen removal performance and analyzed the microbial community, including the abundance of nitrogen metabolism functional genes. Results indicated that the A^2^/O system had higher values for genes associated with nitrification and denitrification than the SBR process (Baeza et al., 2002; Chen ZhaoBo et al., 2011). Thus, understanding the composition and activity of nitrogen metabolism functional genes in different WWTP types is important for optimizing WWTP processes and improving nitrogen removal efficiency. It can help identify the key microbial populations involved in nitrogen transformation and guide the design and operation of WWTPs to achieve effective nitrogen removal and minimize environmental impacts (Holmes et al., 2019; Xu et al., 2022).

## 5 Conclusion

The effects of different processes, locations, and seasons on the nitrogen removal microbes and functional genes in WWTPs were investigated. Seasonal variations or differences in influent water quality had a greater effect on the assembly of microbiomes in WWTPs than location differences, and location differences had a greater effect than process differences. The absolute abundance of AOB *amoA* varied significantly over seasons due to temperature changes. Among NOB, no significant differences were found in *Nitrobacter nxrA* in different processes, geographic locations, and seasons. Interestingly, the absolute abundance of *Nitrobacter nxrB* in AS was higher than that of *Nitrospira*, which could be attributed to differences in influent water quality or operating conditions in sewage treatment plants. The difference in absolute abundance between *nirS* and *nirK* in different locations and seasons was insignificant. The absolute abundance of *nosZ* in DOD was generally higher than that in A^2^/O, and the absolute abundance of *nosZ* in the WWTP of Wuhan (T) was higher than that of the WWTP of Xi’an (B). There was variation in the absolute abundance of anammox 16S rRNA gene between different processes, locations, and seasons, which could be attributed to the sensitivity of functional microbes to DO. Bacteria in terms of the AOB 16S rRNA gene were sensitive to pH, whereas the communities corresponding to AOB *amoA* were more sensitive to temperature proving that the absolute abundance of the anammox 16S rRNA gene in anaerobic AOB may be affected by temperature changes and was hardly affected by seasonal differences. Comprehensive analysis of nitrogen metabolism functional microbes and genes revealed that nitrogen metabolism functional genes *nirS* and *Nitrobacter nxrB* in AS could be related to the unidentified genus of *Flavobacteriales* and *Candidatus Competibacter* as the main nitrogen removal functional microbes, whereas nitrogen metabolism functional genes *nirK* and *Nitrospira nxrB* could be associated with the unidentified genus of *Nitrospiraceae* as the major denitrification functional microbes, which may play significant ecological roles in different niches. This study provides critical insights into the seasonal and geographic factors affecting nitrogen removal processes in WWTPs. By understanding these influences, treatment plants can implement targeted strategies to enhance nitrogen removal efficiency and meet regulatory standards, minimizing the environmental impact of wastewater discharge.

## Data Availability

The datasets presented in this study can be found in online repositories. The names of the repository/repositories and accession number(s) can be found in the article/Supplementary material.
