# Supplementary material for: Comparative analyses on nitrogen removal microbes and functional genes within anaerobic–anoxic–oxic and deoxidation ditch sewage-treating processes in Wuhan and Xi’an cities, China
Source: Front Microbiol. 2024 Oct 30;15:1498681. doi: 10.3389/fmicb.2024.1498681 (PMC11557530; doi:10.3389/fmicb.2024.1498681)
Supplement: Supplementary file 1 [file Data_Sheet_1.docx]

**Supplemental figures:**

**Supplemental figure S1.
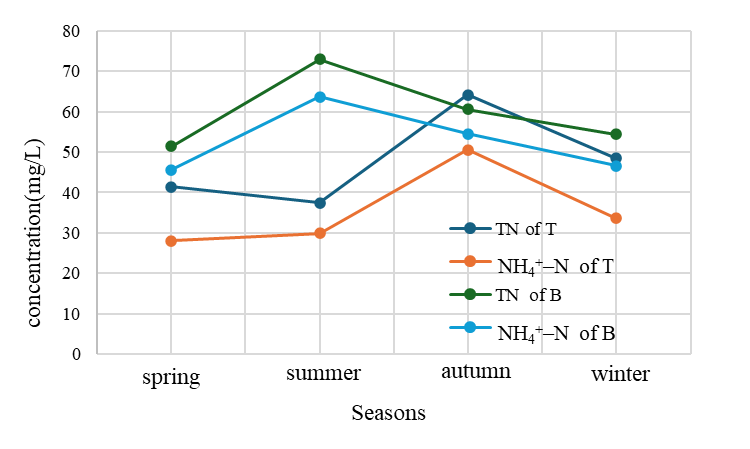
**The concentration of TN and NH_4_^+^–N in the different groups in different seasons.

**Supplemental figure S2.** Rarefaction curves based on the 16S rRNA gene amplicons sequencing. The OTUs were defined of 3% dissimilarity.


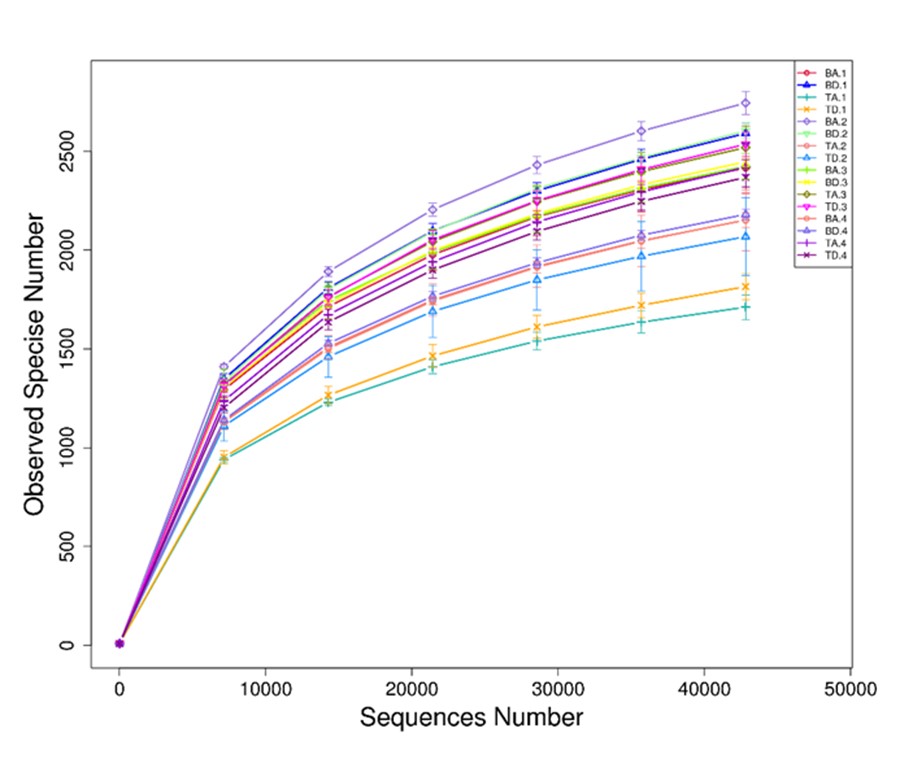


**Supplemental figure S3.** Operational taxonomic unit (OTU) analysis in the different groups; (A) flower diagram showing the shared and unique OTUs in groups from WWTP B; (B) flower diagram showing the shared and unique OTUs in groups from WWTP T.

(A)


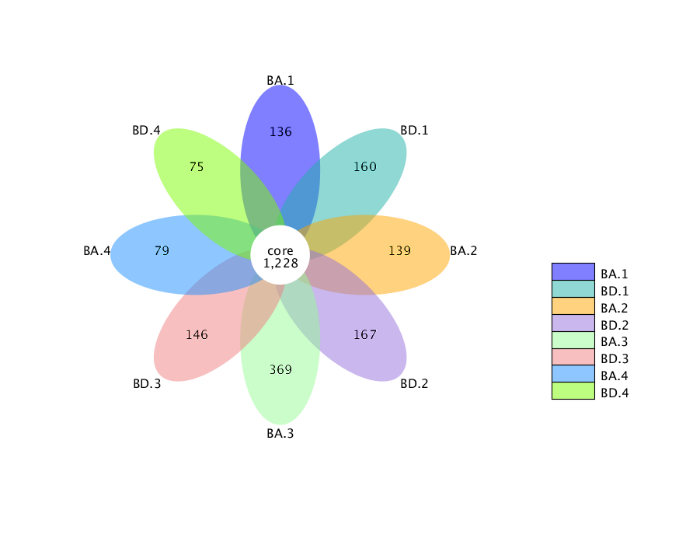


(B)


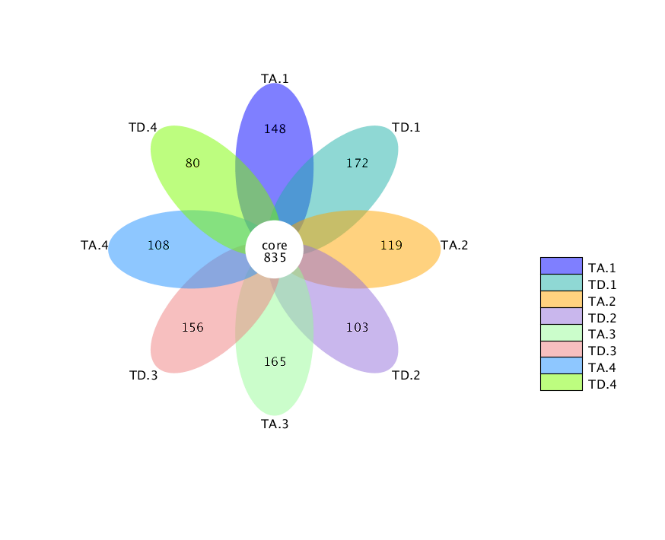


(A)
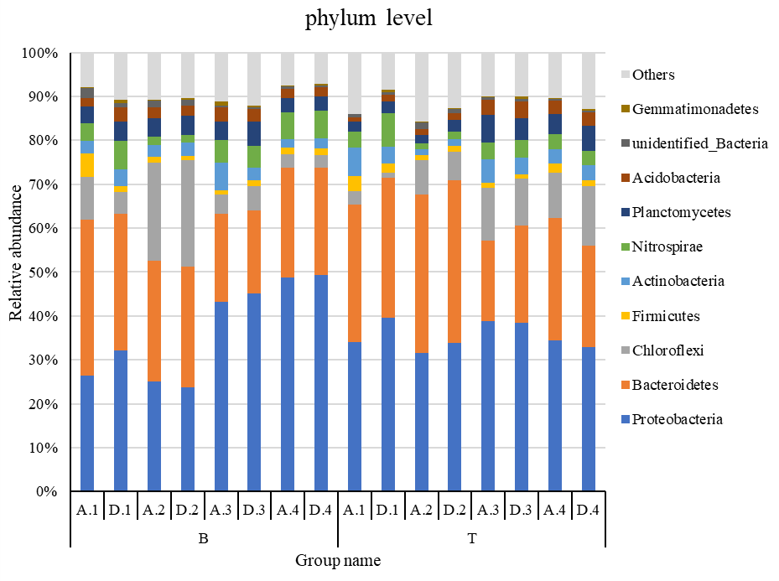


(B)
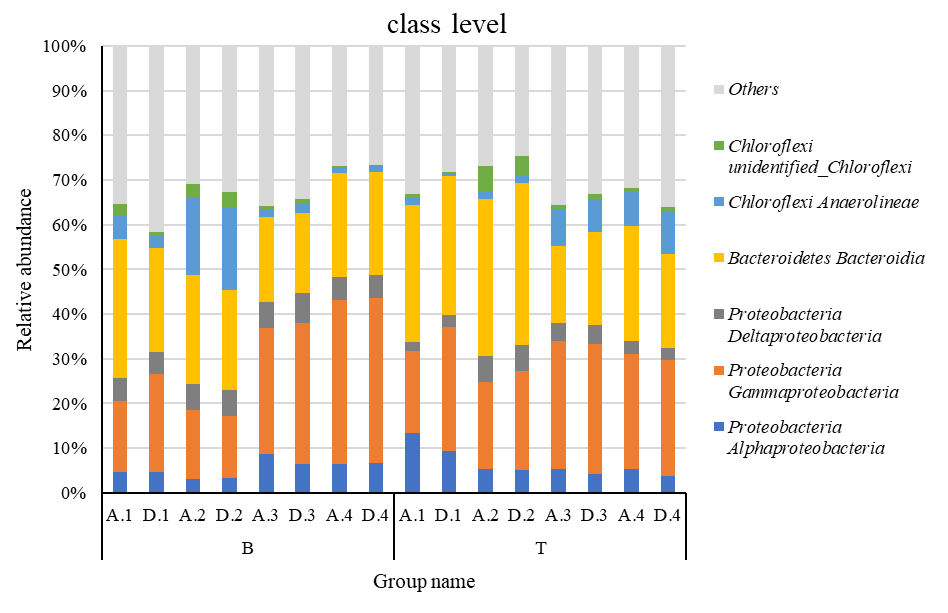


(C)
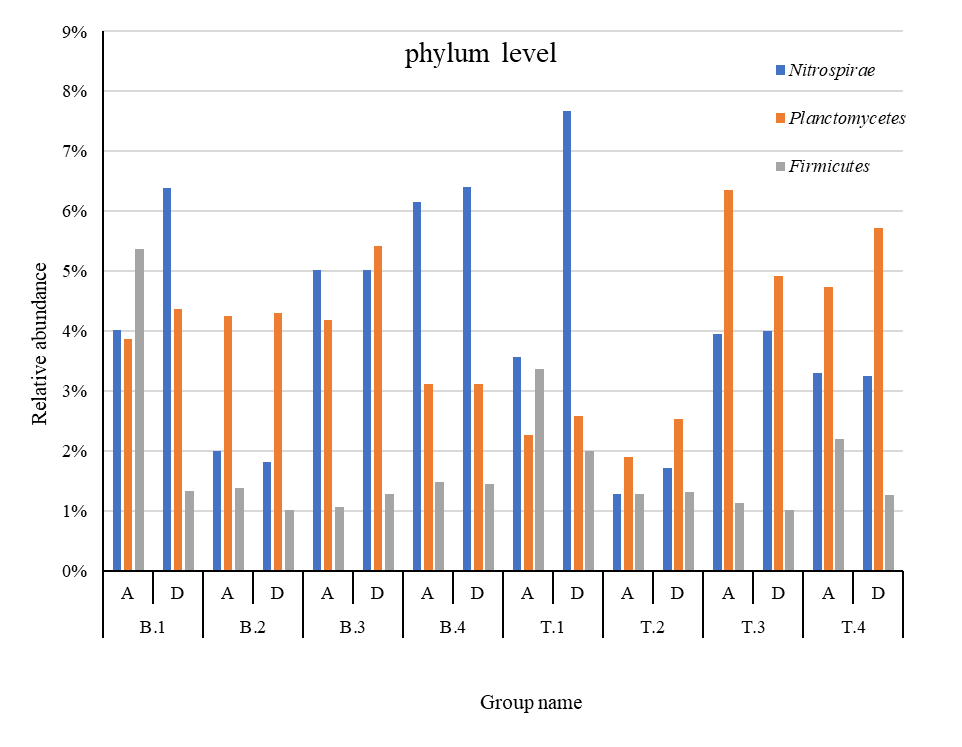


(D)
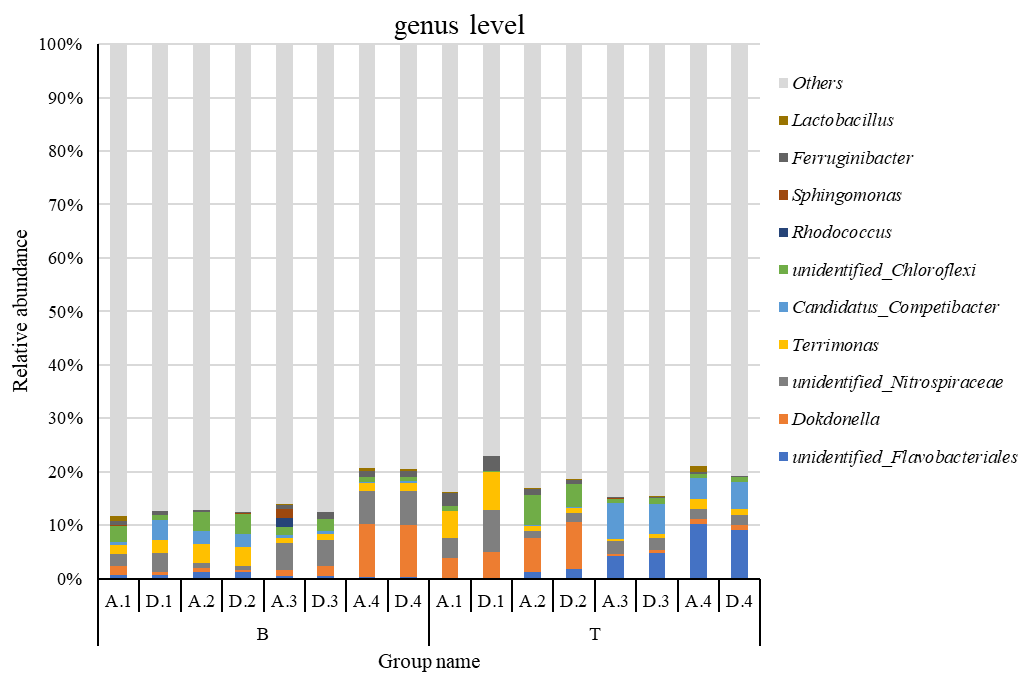


(E)


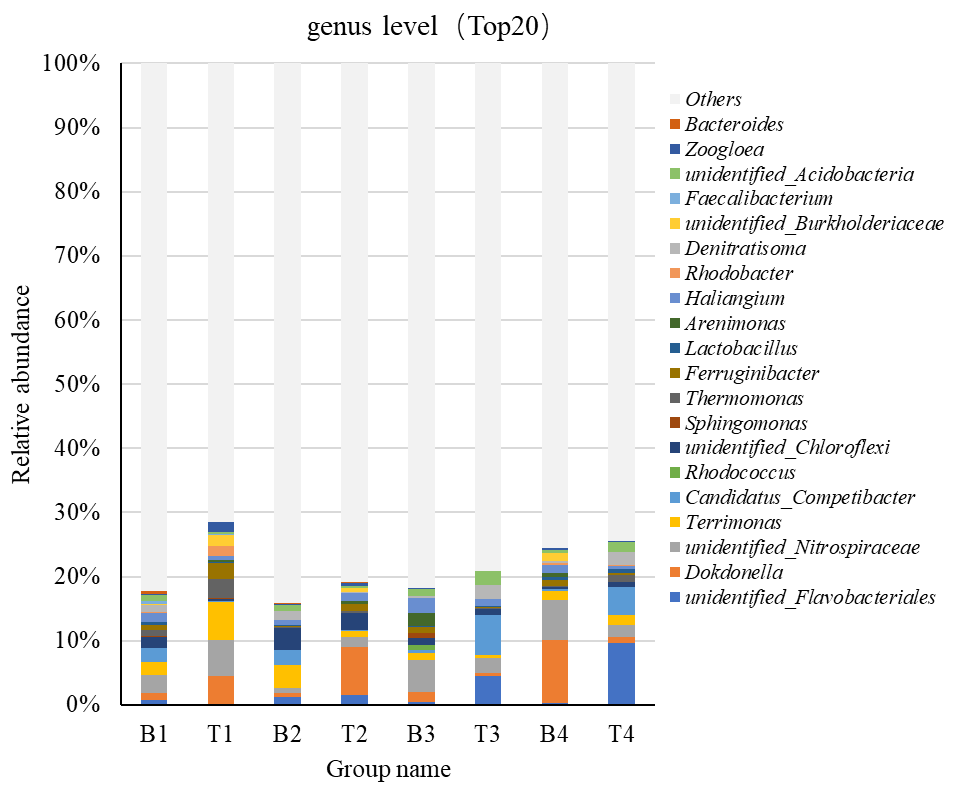


**Supplemental figure S4.** Comparison of microbial community at phylum and genus levels. T and B representing Wuhan and Xi'an respectively; A and D represents A^2^/O and DOD processes respectively; 1, 2, 3 and 4 respectively represent the four seasons of spring, summer, autumn and winter.(A) relative abundance of dominant phylum in each group;(B) relative abundance of class-level composition within the dominant phylum in each group;(C) relative abundance of dominant phylum showing differences between A2/O and DOD processes in WWTPs B and T; (D) relative abundance of the top 10 dominant genera in each group; (E) relative abundance of the top 10 at the phylum level grouped in seasons of WWTPs B and T.

(A)


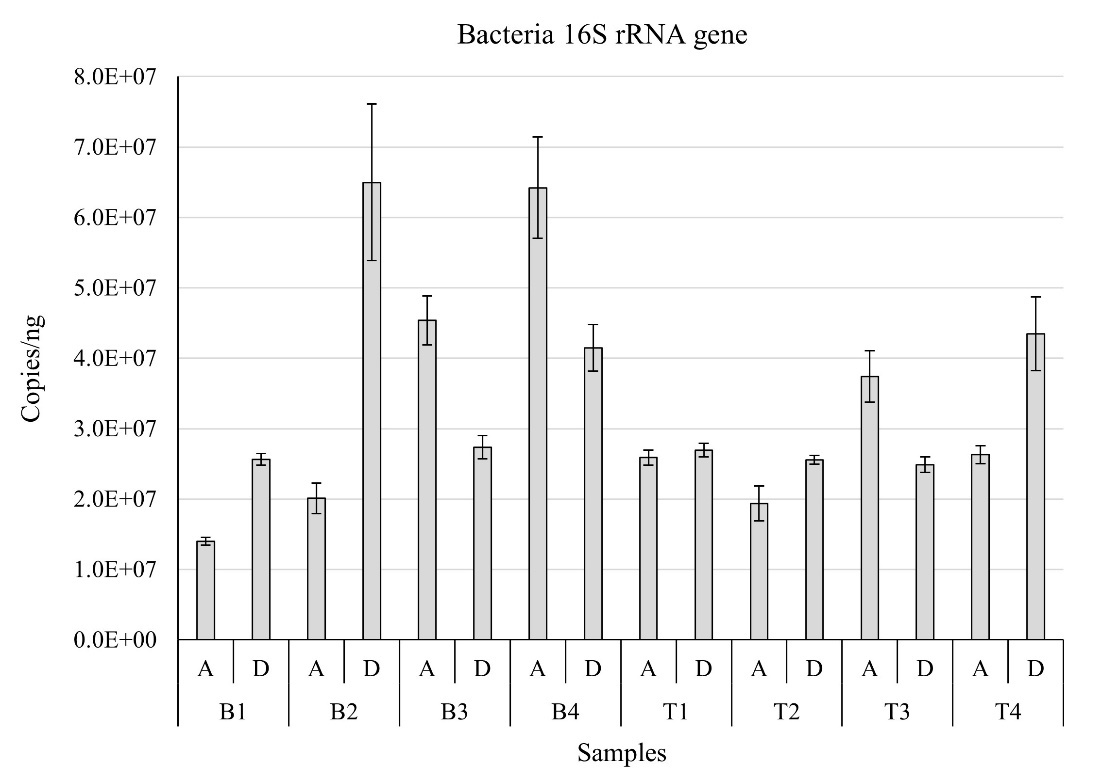


(B)


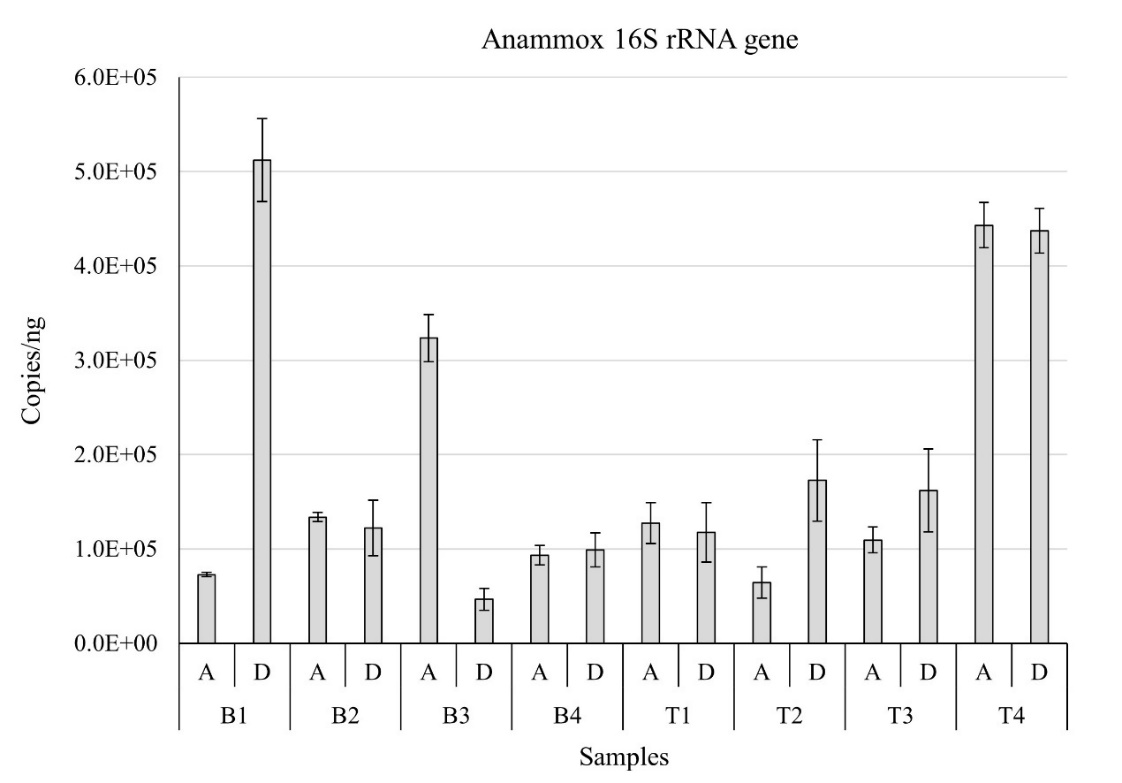


(C)


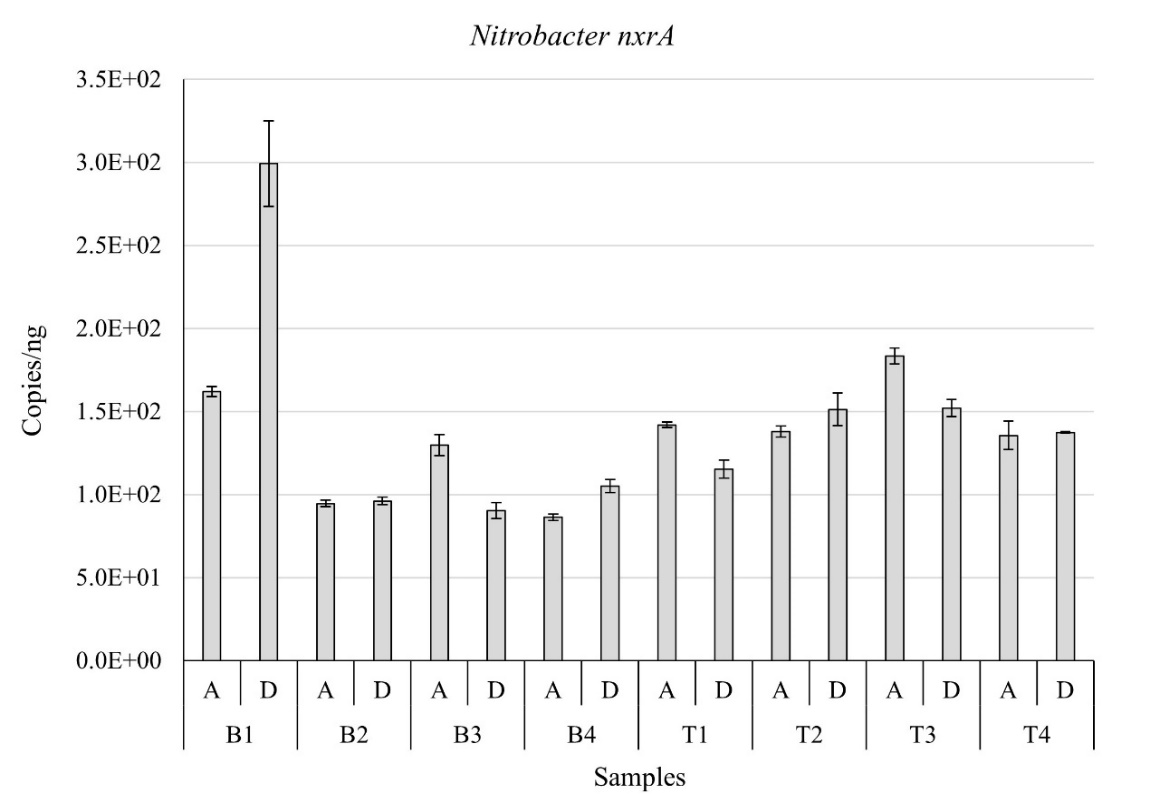


**Supplemental figure S5.** Comparison of absolute abundance of bacterial 16S rRNA gene (A), *Anammox* 16S rRNA gene (B) and *Nitrobacter nxrA* gene (C) in different seasons and processes of WWTPs B and T.
